# Editorial: Genetically determined epilepsies: Perspectives in the era of precision medicine

**DOI:** 10.3389/fneur.2022.1036846

**Published:** 2022-10-11

**Authors:** Mario Mastrangelo, Vincenzo Salpietro, Joseph Sullivan

**Affiliations:** ^1^Division of Child and Adolescent Neurology and Psychiatry, Department of Human Neuroscience, Sapienza University of Rome, Rome, Italy; ^2^Department of Neuroscience and Mental Health, Umberto I-Polyclinic Rome, Rome, Italy; ^3^Department of Pediatrics, University of L'Aquila, L'Aquila, Italy; ^4^Department of Neuromuscular Diseases, University College London, London, United Kingdom; ^5^Division of Epilepsy, Department of Neurology, University of California, San Francisco, San Francisco, CA, United States

**Keywords:** genetic epilepsies, next generation sequencing, neurodevelopmental disorders, copy number variants, children

The complex landscape of genetic epilepsies was largely expanded in the last decade. To date, a specific genetic etiology can be identified for up to 40% of severe early onset drug-resistant developmental and epileptic encephalopathies, but also for milder seizure phenotypes with no significant comorbidities (1). Around 7,000 single genes and more than 400 different chromosomal imbalances with a presumed pathogenic role were identified and reported in the literature (1–3). More than 210 genes were directly implicated in peculiar epilepsy phenotypes, even though eight genes accounted for most of cases in a recent epidemiological survey in United Kingdom (1–3). These numbers mirror a huge progress since the first association between *CHRNA4* and nocturnal frontal lobe epilepsy in 1995 (4).

The aim of this Research Topic is to offer a real-world panorama on the field of genetic epileptology. Several experts, from different countries all over the world, provided new updated data and interesting analysis of the correlated clinical and therapeutic perspectives (https://www.frontiersin.org/research-topics/19762).

The ease of obtaining next generation sequencing (NGS) in clinical practice has resulted in significant improvement in the diagnostic yield with a remarkable increase of etiological diagnosis. This led to a reduction of diagnostic timing and costs, while also allowing for more precise genetic counseling and an ongoing discovery of new disease-causing genes with novel genotype-phenotypes associations (5). The possibility of concurrently analyzing a wider group of disease-causing genes and the faster gene-sequencing raises other clinical questions that must be considered including (but not limited to): (i) the availability of a large amount of data that often complicate genotype-phenotype correlations; (ii) the frequent detection of variants of uncertain significance; (iii) the frequent need for functional studies to assess the real pathogenic effect of the detected variants; (iv) a limited epidemiological impact (most of the known disease-causing genes associated with epilepsy accounts for a limited quote of cases).

As more genetic etiologies are going to be discovered, this requires parallel changes in the clinical approach to patients with epilepsy as careful phenotyping is critical for the correct interpretation of molecular genetic data. Further practical implications include heterogeneity with regard to different clinical phenotypes resulting from variants of the same genes (e.g., *SCN2A* causes both familial benign neonatal infantile epilepsy and a severe epileptic encephalopathy; *KCNQ2* was initially associated with familial benign neonatal seizures and, subsequently, with an early onset epileptic encephalopathy) or similar clinical syndromes caused by different genes (e.g., Dravet syndrome can be caused by pathogenic variants in *SCN1A, PCDH19, STXBP1* or *GABRA1*). The additional role of several genetic modifiers in defining increased susceptibility to epileptogenic processes added complexity to the polygenic etiological basis of different epilepsy phenotypes (5).

The impact of a molecular genetic diagnosis on treatment choices has resulted in different possible approaches (e.g., avoiding, stopping, or initiating specific antiseizure medications or ketogenic diet; addressing or avoiding surgery) in 12 and 80% of the cases significant (6). Unlike other neurological diseases, gene therapy for genetic epilepsies still has a number of obstacles to overcome (e.g., excessive size of some epilepsy genes, such as *SCN1A*, for common viral vectors; immunogenicity of CRISPR-Cas-9 system; difficulties to control gene dosing) and the ambitious target to alter the natural history and improve upon other non-seizure related comorbidities (7).

The advances in making a genetic diagnosis are likely to lead to changes in how were approached patient diagnoses and management such as:

a) a gradual shift from classical syndromic classifications of epilepsies to a nosology based on molecular genetic etiology (e.g., *SCN2A*-encephalopathy, KCNQ2 encephalopathy etc.);
b) increasing knowledge regarding the natural history and longer-term outcomes of monogenic and copy-number variation-related epilepsy disorders by collecting more data on adult patients (both in terms of prolonged follow-up since infancy and as novel diagnosis in adult neurology settings).
c) an increasing number of treatments tailored to the molecular genetic defects (e.g., sodium channel blockers for *SCN2A* and *SCN8A* encephalopathy; ezogabine for KCNQ2/3 defects; quinidine for *KCNT1* variants; everolimus for m-TORopathies; ketogenic diet for GLUT-1 deficiency; etc.).

The understanding of these advances is pivotal to more defined phenotyping and timely diagnosis (e.g., next generation sequencing as gold standard for early genetic diagnosis, links between international clinical reference networks, online databases or *ad-hoc* registries) and increasing collaboration between basic scientists and clinical researchers (e.g., early availability of functional studies and advanced tools for designing oriented therapeutic trials) to improve upon our treatment approaches and outcomes that extends beyond treating just seizures and rather treats the entire genetic epilepsy condition.

## Author contributions

MM projected the Research Topic, coordinated the process, and wrote the first draft of the editorial. VS and JS coordinated the revision process of the papers and revised the draft of the editorial. All authors contributed to the article and approved the submitted version.

## Conflict of interest

The authors declare that the research was conducted in the absence of any commercial or financial relationships that could be construed as a potential conflict of interest.

## Publisher's note

All claims expressed in this article are solely those of the authors and do not necessarily represent those of their affiliated organizations, or those of the publisher, the editors and the reviewers. Any product that may be evaluated in this article, or claim that may be made by its manufacturer, is not guaranteed or endorsed by the publisher.
